# Recent advances in integrated optical directed logic operations for high performance optical computing: a review

**DOI:** 10.1007/s12200-022-00001-y

**Published:** 2022-03-28

**Authors:** Ciyuan Qiu, Huifu Xiao, Liheng Wang, Yonghui Tian

**Affiliations:** 1 State Key Lab of Advanced Optical Communication Systems and Networks, Department of Electronic Engineering, Shanghai Jiao Tong University, Shanghai, 200240 China; 2 Institute of Microelectronics and Key Laboratory for Magnetism and Materials of Ministry of Education, School of Physical Science and Technology, Lanzhou University, Lanzhou, 730000 China

**Keywords:** Optical computing, Directed logic (DL), Optical switch

## Abstract

Optical directed logic (DL) is a novel logic operation scheme that employs electrical signals as operands to control the working states of optical switches to perform the logic functions. This review first provides an overview of the concept and working principle of DL. The developing trends of DL computing are then discussed in detail, including the fundamental optical DL gates, combinational optical DL operations, reconfigurable logic computing, low power optical logic computing, and programmable photonic network. The concluding remarks provide an outlook on the DL future development and its impacts in optical computing.

**Graphical Abstract:**

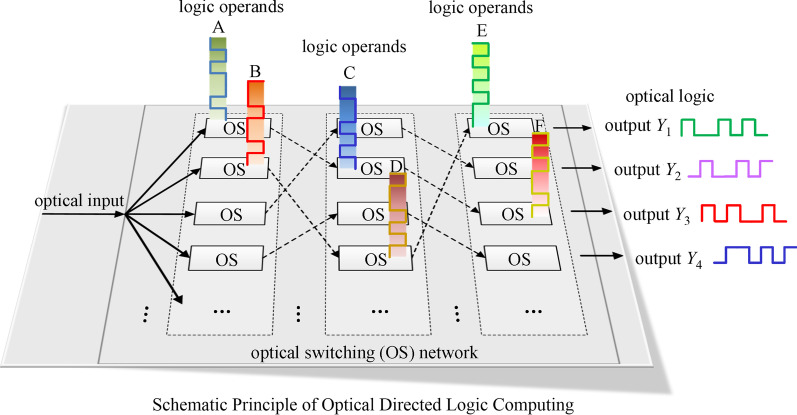

## Introduction

Logic operation is one of the most fundamental and crucial functions of integrated circuits. However, in traditional electronic circuit, the fundamental logic gates performing Boolean operations are cascaded to implement computing and information processing. Consequently, the delay of each logic gate in switching processes accumulates to affect the performance metrics of the circuit as a whole [1, 2]. On the other hand, Moore’s Law, a rule of thumb that dominates computing [3], is faced with increasingly difficult challenges as the nanofabrication technique comes to a several-nanometer limit, where quantum uncertainties governs electron behavior, making transistors unreliable [4, 5]. Thus, it is urgent for researchers to develop a new mechanism or carrier to realize logic computing.

Light is a promising carrier candidate to implement logic computing, since photon possesses many distinguished advantages, including high transmission speed, low latency, high frequency, and large bandwidth [6–9]. Silicon photonics has been poised to revolutionize traditional electrical information processing due to their low loss of silicon and silicon dioxide in the C-band, and the arguable amenability of the low-cost high-volume manufacturing process of integrated devices based on the silicon-on-insulator (SOI) platform [10–12]. Generally, all-optical logic and directed logic (DL) are the two main schemes of silicon-based on-chip optical logic computing [13, 14].

In all-optical logic, the operands and operation results are all photons [15, 16], thus all-optical logic computing can be implemented by a semiconductor optical amplifier (SOA) [17, 18], micro-ring resonator (MRR) [13, 19, 20], photonic crystal [21–24], and plasmon waveguide [25–27]. Although a high operation speed can be achieved in all-optical logic, a “high power requirement” is usually inevitable for input light to excite the two-photon absorption or four-wave mixing effect of silicon waveguide [28–31] and the propagation loss for plasmon waveguide is relatively large [25–27], all of which are inconvenient for large-scale integration.

In optical DL, the operands are electrons, while the operation results are photons. Electrical signals are employed to control the status of optical switches in an optical network to implement the logic functions [14, 32]. The electrical signals applied in directed logic are parallel, so that all the switches can be implemented at the same time. The computed results are transmitted by photon on a chip to maintain a high computing speed and prevent the switching latency from accumulating [33]. No power source is required for DL computing. More importantly, light can be high-frequency modulated and there are many multiplexing techniques for DL to be parallel processed in a large capacity [34]. By harnessing the advantages of electrons and photons (easy to control/store and high in performance, respectively) in optical computing, the DL mechanism is believed to pave the way for realizing the next-generation computer named the “electronic-photonic digital computer” [35]. While tremendous work has been performed on DL in recent years due to its great significance in optical computing [32, 33, 35, 36], there is still a lack of discussion regarding the future development and advancement of DL computing.

In this paper, we present a review of the current development of integrated optical DL operations on SOI platform and indicate the potential trend of integrated optical DL operations in the near future. This paper is organized as follows: Sect. 2 focuses on the working principle of optical DL functions and provides an overview of the current trends of optical DL, including fundamental DL gates and combinational logic gates. In Sect. 3, a novel DL mechanism named reconfigurable DL for arbitrary logic operations is explored and three performance approaches are discussed. In Sect. 4, the low-power optical computing and its building blocks are discussed. In Sect. 5, two types of programmable photonic networks, both efficient methods for complex optical DL computing, are reviewed. Section 6 is the conclusion.

## Optical directed logic (DL) operations

### Principle of optical DL

**Fig. 1:**
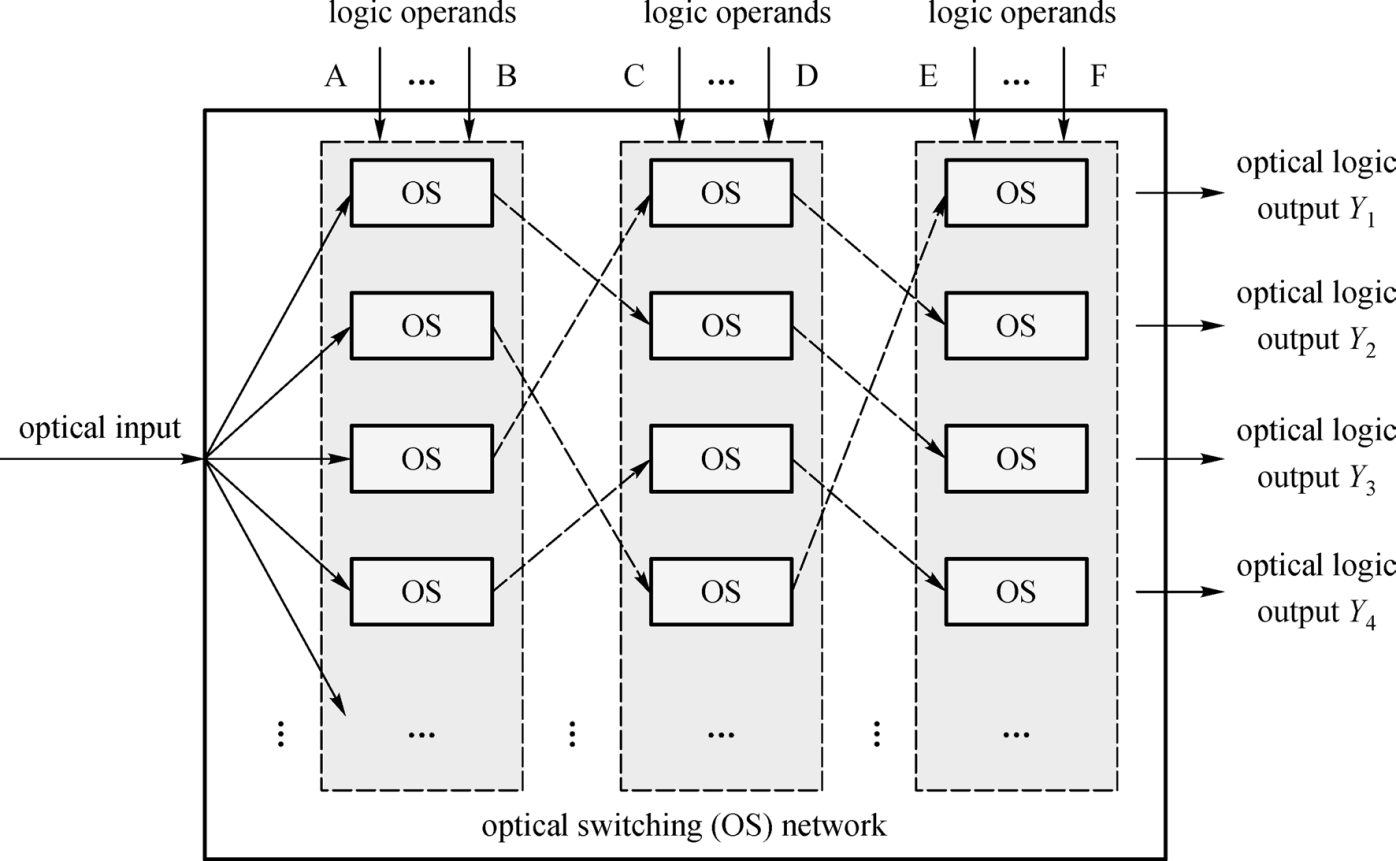
Working principle of optical directed logic composed of optical switches (OS: optical switch)

In 2007, Hardy and Shamir proposed the first optical inspired DL architecture [14], the principle of which is shown in Fig. 1. The architecture of DL circuit is composed of an optical switching network. The logic operands are applied on the optical switches to control their working states (“cross”, “bar”, “block” or “pass”) in parallel and direct the input continuous light to different optical paths. Ideally, the switching processes can be implemented simultaneously since the logic operands are independent and the next stage switching operation can proceed without waiting for the previous switching results due to light-speed transmission in the network. Whether a high level of optical power is detected in a specific output port is regarded as an optical logic result of “1” or “0”. Therefore, the parallel input electrical operands can be computed without delay accumulations and the computing results are in the form of light. In other words, the DL scheme merges the advantages of electrons and photons while avoiding their drawbacks, which inserts the high performance of light into logic operation to improve its performance metrics.

According to the principle shown in Fig. 1, the computation of optical logic function can be performed by one or more switches. There are many kinds of optical switches that can be adopted to form the optical switching network on SOI platform, such as the Mach–Zehnder interferometer (MZI) [37–40], MRR [41–44], and micro-disk resonator (MDR) [45–48]. Among them, MZI is an interference device, while MRR and MDR are resonant devices. The bandwidth of MZI can be very large to the point where it covers the entire C band (1525–1565 nm) and is relatively insensitive to temperature change and fabrication deviation. The device size and power consumption are relatively large as well [49, 50]. On the other hand, the device size and power consumption of MRR are very small in comparison to MZI. Due to its character of resonance, MRR possesses several excellent properties, including filtering and wavelength tuning [51–53], though its bandwidth is small and temperature sensitivity is relatively high. The device size and power consumption of MDR can be even smaller than MRR [54, 55], but it is much more sensitive to temperature change. Given the information above, one can choose the appropriate switches to form the optical switching network for DL according specific application requirements.

### Fundamental optical DL gates

Fundamental logic functions, including NOT, AND/NAND, OR/NOR and XOR/XNOR, are vital in DL optical computing since they can be combined and cascaded to implement any logic computation function. Various fundamental DL gates with different topological structures have been demonstrated since DL was first proposed. This sub-section is to review the progression of fundamental DL gates.

**Fig. 2:**
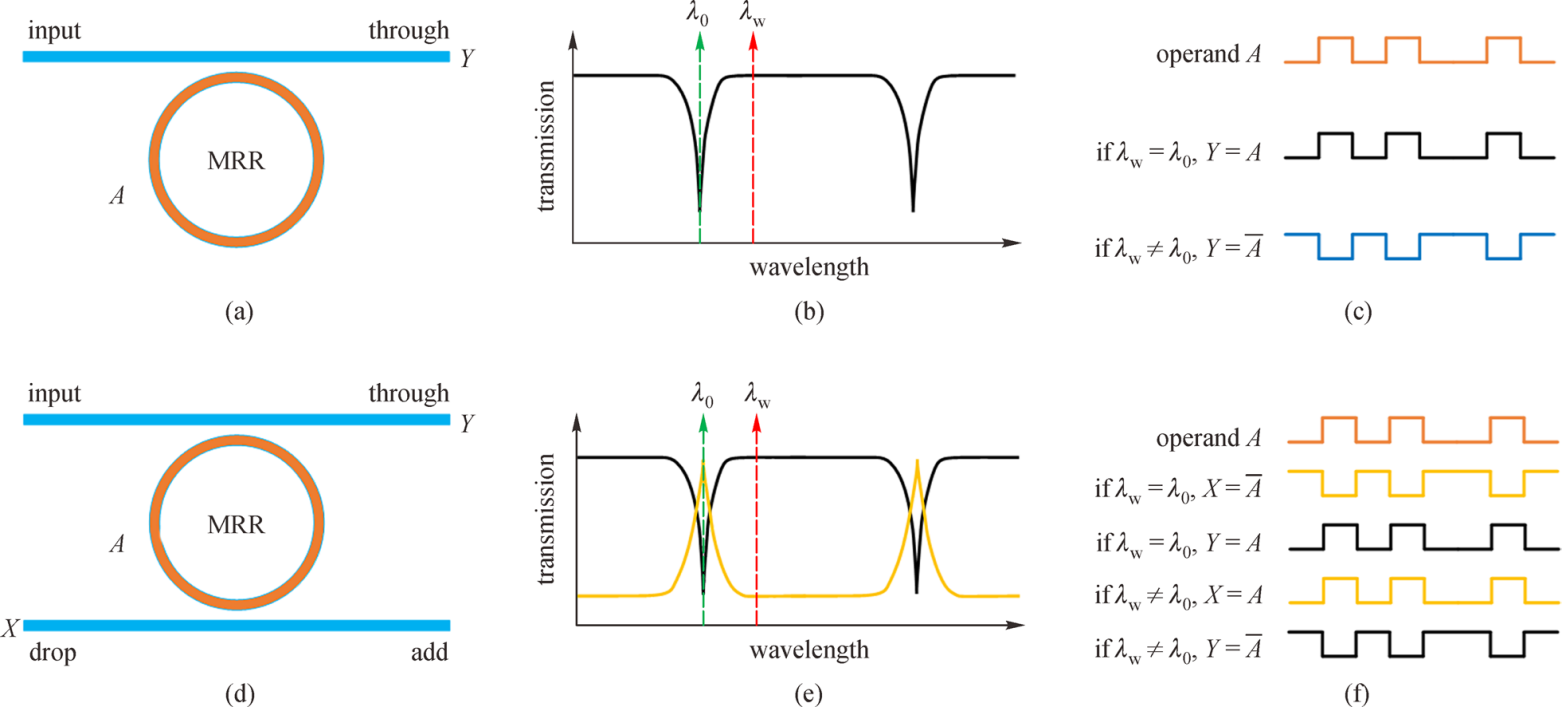
Implementation of NOT operation by MRR. **a**–**c** Structure, transmission spectrum, and logic operations of MRR with single waveguide, if the working wavelength *λ*_w_ and the resonant wavelength *λ*_0_ are the same, the MRR can perform NOT operation. **d**–**f** Structure, transmission spectrum, and logic operations of MRR with double waveguides, if *λ*_w_ is the same as *λ*_0_, the NOT operation can be obtained in the Drop port, while if *λ*_w_ is not the same as *λ*_0_, the NOT operation can be obtained in the Through port

Among all the fundamental logic functions, the logic operation of NOT, the key element for optical inverter [56], universal gate [57], shift register [58], and some non-trivial all-optical functionalities [59] can be implemented by just a single optical switch. As shown in Fig. 2, taking MRR with a single waveguide as an example, the NOT operation can be obtained when the working wavelength, *λ*_w_, is not the same as the resonant wavelength, *λ*_0_, thus the input electrical operand 0 is converted to an optical logic 1 because light is off-resonance at *λ*_w_, and the input electrical operand 1 is converted to an optical logic 0 because light is on-resonance at *λ*_w_. In the case of MRR with double waveguides, the NOT operation can be obtained in either the Drop or Through port, which is determined by whether *λ*_w_ is the same as *λ*_0_ or not. The phenomenon of different working wavelengths resulting in different logic operations allows for high versatility of the optical DL circuit.

**Fig. 3:**
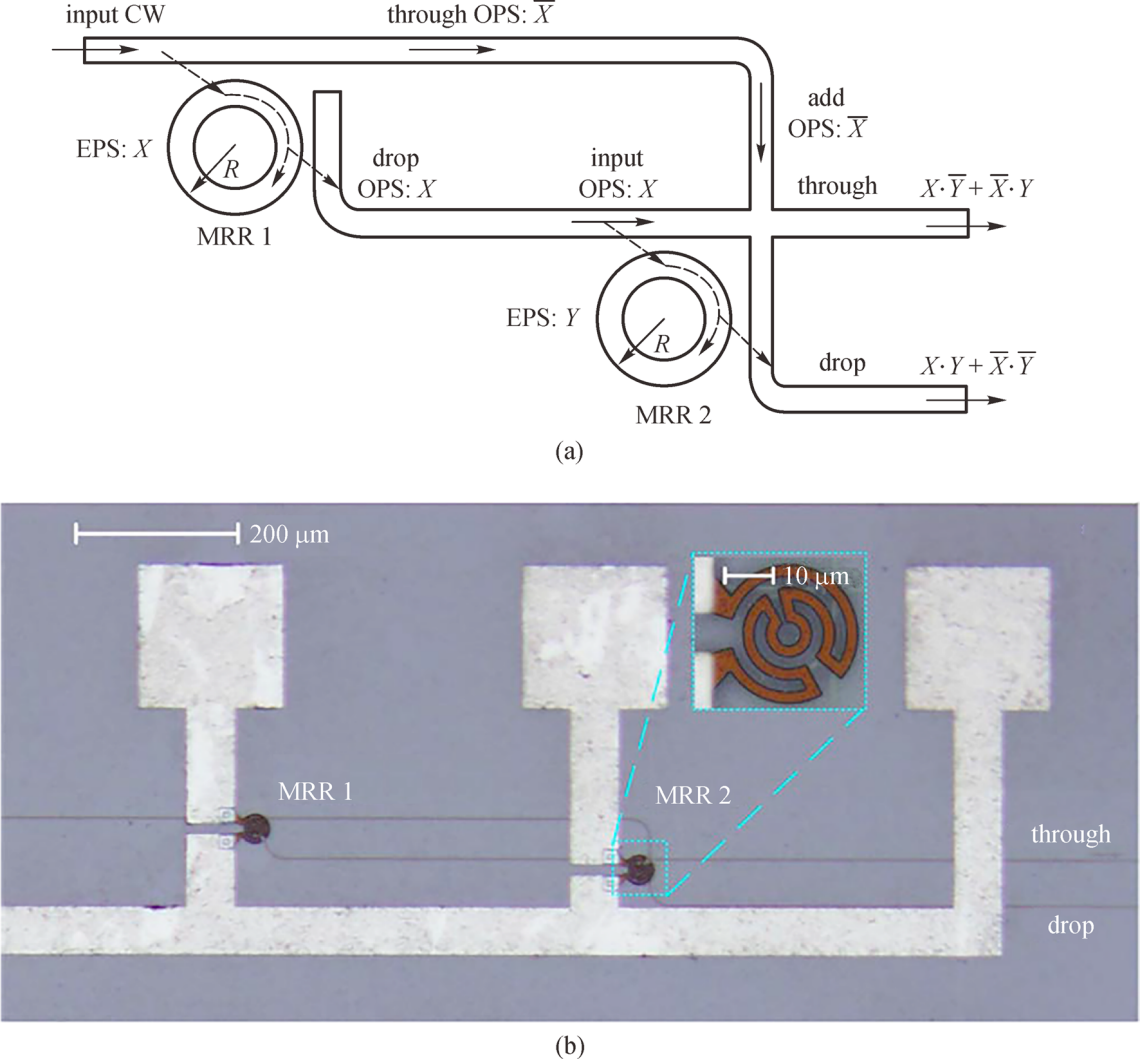
Optical circuit utilized as optical XOR/XNOR calculator. **a** Schematic structure and **b** micrograph of the circuit. MRR: micro-ring resonator, CW: continuous wave, EPS: electrical pulse sequence, OPS: optical pulse sequence. Reprinted with permission from Ref. [36]. Copyright 2010, The Optical Society

The XOR/XNOR operation is indispensable and can be applied in many fields, such as label processing, parity checking, data encryption/decryption, and pseudorandom number generation. In 2010, Dr. Lei Zhang and Prof. Lin Yang from the Institute of Semiconductors in Chinese Academy of Sciences proposed and experimentally demonstrated a DL architecture to perform XOR and XNOR operations [36], the schematic structure and micro-graph of which is shown in Fig. 3. In that device, MRR is on-resonance at working wavelength *λ*_w_ when the voltage applied is at high level (input operand “1”), and off-resonance when no voltage is applied (input operand “0”). Both thermo-optic and plasma dispersion effects of silicon were adopted to tune the MRRs [60, 61]. When the logic operands applied to MRR1 and MRR2 are all 0 or 1 (*X* = 0 and *Y* = 0 or *X* = 1 and *Y* = 1), the input continuous wave (CW) is directed to the Drop port of MRR2, and when one of the operands *X* and *Y* is 1 (*X* = 0 and *Y* = 1 or *X* = 1 and *Y* = 0), the input CW is directed to Through port of MRR2. This means that the logic operation of XOR can be obtained in Through port and XNOR can be obtained in Drop port simultaneously, indicating that the DL holds an inherently distinguished advantage of computing both a function and its inverse simultaneously. However, a waveguide crossing exists in the device structure of the proposed XOR/XNOR calculator, which deteriorates the performance of device in insertion loss and crosstalk. To eliminate the waveguide crossing, several works have been proposed and carried out using different U-shaped waveguides [62–65].

**Fig. 4:**
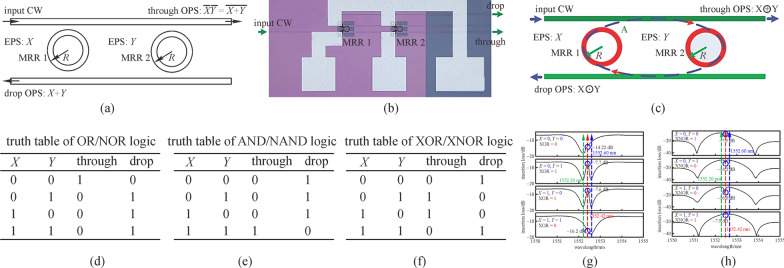
Optical circuit utilized to demonstrate optical OR/NOR, AND/NAND, and XOR/XNOR operations. **a** Schematic structure and **b** micrograph of the circuit. **c** Schematic principle to implement XOR/XNOR (X⊙Y and X⊕Y), in which the photonic analog to electromagnetically induced transparency (EIT) phenomenon, also known as coupled-resonator-induced transparency (CRIT) is utilized. **d**–**f** Truth tables that the circuit can implement. **g** and **h** Static response spectra of the circuit based on CRIT effect. **a** and **b** Reprinted with permission from Ref. [66]. Copyright 2011, The Optical Society. **c**, **g**, and **h** Reprinted with permission from Ref. [69]. Copyright 2013, Wiley

By cascading optical MRRs, the operations of AND/NAND and OR/NOR can also be implemented. In 2011, a photonic circuit composed of two parallel MRRs with two waveguides was proposed to implement OR/NOR and AND/NAND logic functions [66], as shown in Fig. 4. The MRRs are first set to be on-resonance when operands “1” are applied and off-resonance when operands “0” are applied. In this working mode, the logic functions of OR (*X* + *Y*) at the Drop port and NOR ($\overline{X + Y}$) at the Through port were obtained. Then, once the MRRs in the same structure are set to be on-resonance when operands “0” are applied and off-resonance when operands “1” are applied, the logic functions of AND (*X·Y*) and NAND ($\overline{X\cdot Y }$) can be obtained at the Drop and Through ports respectively. In 2014, this device was demonstrated to achieve a working speed of 100 Mbps and 10 Gbps by adopting forward-biased p-i-n diodes and reverse-biased p-n junctions to tune the MRRs respectively [67, 68]. The structure can also implement XOR/XNOR operations [69] by utilizing the coupled-resonator-induced transparency (CRIT) phenomenon [70], of which the spectra are shown in Fig. 4g and h. One advantage for using the CRIT effect to implement XOR and XNOR operations is that the dynamic power for performing logic computing can be greatly reduced due to the small driving voltages needed to generate a resonance difference.

In addition to MRR, MZI can also be utilized to perform logic operations. In 2017, a XOR/XNOR optical logic circuit based on silicon MZI was proposed and demonstrated [71]. p-n junctions were embedded into two arms of the MZI to implement a high-speed modulation and a thermal heater was adopted to control the original phase difference between the two arms. Since XOR and XNOR are reverse logic functions, once the device is optically biased at the minimum or maximum transmission points by tuning the heater on one arm, XOR or XNOR operations can be performed respectively at a speed up to 20 Gbps.

**Fig. 5:**
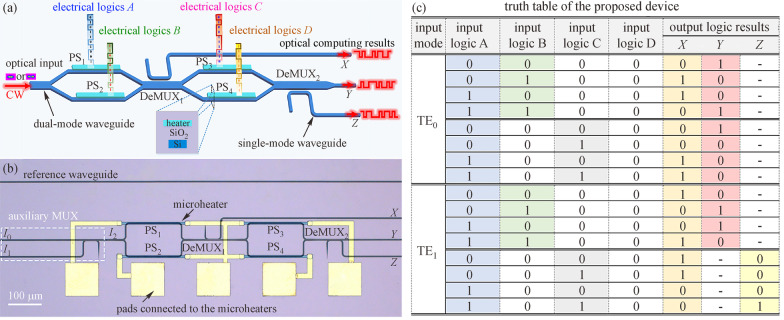
**a** Schematic structure, **b** micrograph, and **c** truth table of the multi-functional logic circuit based on mode selecting property. PS: phase-shifter, DeMUX: mode demultiplexer, MUX: mode multiplexer. **a** and **b** Reprinted with permission from Ref. [72]. Copyright 2021, The Optical Society

Other fundamental logic operations can also be implemented by cascading MZIs. In 2021, Dr. Huifu Xiao and Prof. Yonghui Tian from Lanzhou University proposed and experimentally demonstrated an optical circuit that can be reconfigured to implement six DL operations based on the optical mode selecting property [72], structure, micrograph, and truth table of which are shown in Fig. 5. In that device, input logic “1” means that a proper voltage is applied on one arm to produce an additional phase accumulation of π, and input logic “0” corresponds to no additional phase accumulation. By employing the property that different modes have different propagation constants and transmission features in one waveguide, the logic functions of XOR, XNOR, BUFFER, NOR, NOT, and AND were successfully demonstrated with optical signal-to-noise ratios larger than 17.6 dB over the entire C band (1525–1565 nm) and dynamical working speed of 10 Kbps. The proposed device was characterized with simple structure, large bandwidth, and high versatility, thus is believed to be a promising candidate for information processing in optical MDM networks.

### Combinational optical DL operations

Combinational logic computing is a type of digital logic operation implemented by Boolean circuits, where the output is a pure function of the present input only. It can be directly utilized to perform Boolean algebra on input signals and stored data, such as encoding/decoding, half-adding/subtracting, comparing, and parity checking. In electrical circuits, the combinational logic operations are usually cascaded by several fundamental logic gates, which would lead to delay accumulation and consequently affect the maximum operational speed of the logic circuit. However, unlike electrical circuits, optical combinational logic functions are usually implemented by an entire optical switching network directly in optical DL [14], thus does not lead to delay accumulation. Various combinational DL operations have been demonstrated [73–84] and recent publications are to be introduced in this sub-section.

**Fig. 6:**
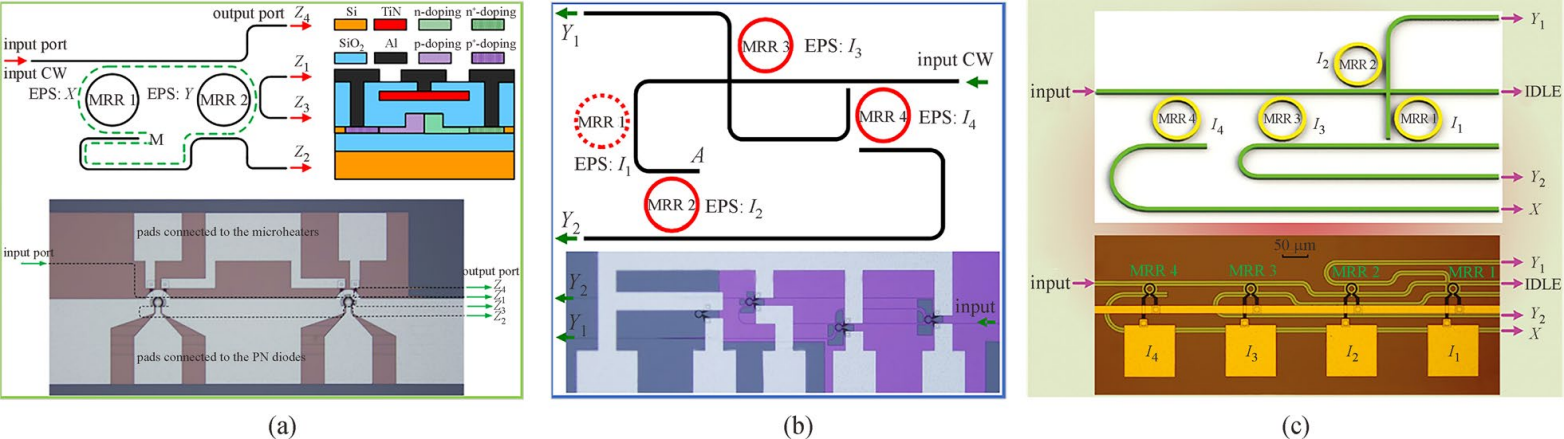
Schematic structures and micrographs of photonic circuits utilized to implement **a** decoding, **b** encoding, and **c** priority encoding, respectively. **a** Reprinted with permission from Ref. [74]. Copyright 2014, The Optical Society. **b** Reprinted with permission from Ref. [75]. Copyright 2011, The Optical Society. **c** Reprinted with permission from Ref. [76]. Copyright 2018, Walter de Gruyter GmbH

Optical decoders and encoders are important devices in optical combinational DL operations, with additional applications in image processing, digital display, and data switching. In 2011, a silicon photonic circuit composed of two MRRs and three waveguides was proposed to implement the decoding operation from two binary logics to four-bit optical logic [73], as shown in Fig. 6a. In that device, a normal resonator and a resonator coupled with three waveguides were adopted in the architectural design. The corresponding working speed was demonstrated up to 1 Gbps by utilizing reverse-biased p-n junction embedded MRRs [74]. In the case of optical encoding, the truth table is the reverse of encoding, but the operand is electron while the optical result is photon in DL, thus the architecture of optical encoding should be different from the one for decoding. Figure 6b is a proposed optical encoder architecture that can encode a four-bit electrical signal into a two-bit binary optical signal [75]. Priority encoder is a special encoder that can identify the priority levels of the request signals and encode them with specific meanings. Due to this characteristic, priority encoders are indispensable for applications in on-chip optical network, control system, emergency responders, etc. In 2018, an on-chip optical priority encoder that can encode a 4-bit electrical signal into a 2-bit optical signal according to priority was proposed and experimentally demonstrated with a working speed of 10 Kbps [76]. The schematic architecture and micrograph of that device, which is composed of four MRRs and four waveguides, are shown in Fig. 6c.

Another two key combinational logic operations for digital optical computing are half-adding and half-subtracting. In 2012, the first DL computing for two-bit half adding operation was implemented by a silicon photonic circuit composed of two MRRs and a multimode interference (MMI) coupler [77]. Considering that the MRR is a power splitter and the MMI is a power combiner in that device, the computed results always have two stages in the Sum port *Y*_2_. An effective method to eliminate this phenomenon is to avoid using MMI in the circuit [78]. Subsequently, a circuit was demonstrated to perform half-subtracting operation [79]. Other important combinational DL devices, including the comparator [80–82], parity checker [83], and Feynman gate [84] were also demonstrated with relatively low working speed for proofs of concepts in recent years. To achieve higher operational speeds, one can employ other advanced modulation schemes, such as the plasma dispersion effect [85, 86], graphene-based Fermi level modulation [87–89], or lithium niobate waveguide [90–92] to modulate the MRRs.

## Reconfigurable DL circuit

In Sect. 2, the recently thriving progress of DL in SOI platform was reviewed. However, the majority of DLs in the reviewed papers have a single fixed function or only a limited number of functions, meaning many types of DL need to be designed to meet all the application requirements for different occasions. Inevitably, this would increase the developing cost and research period. To solve this issue, reconfigurable optical logic schemes were developed since they can perform all combinational logic operations by changing the operation states of optical switches in a pre-designed circuit.

**Fig. 7:**
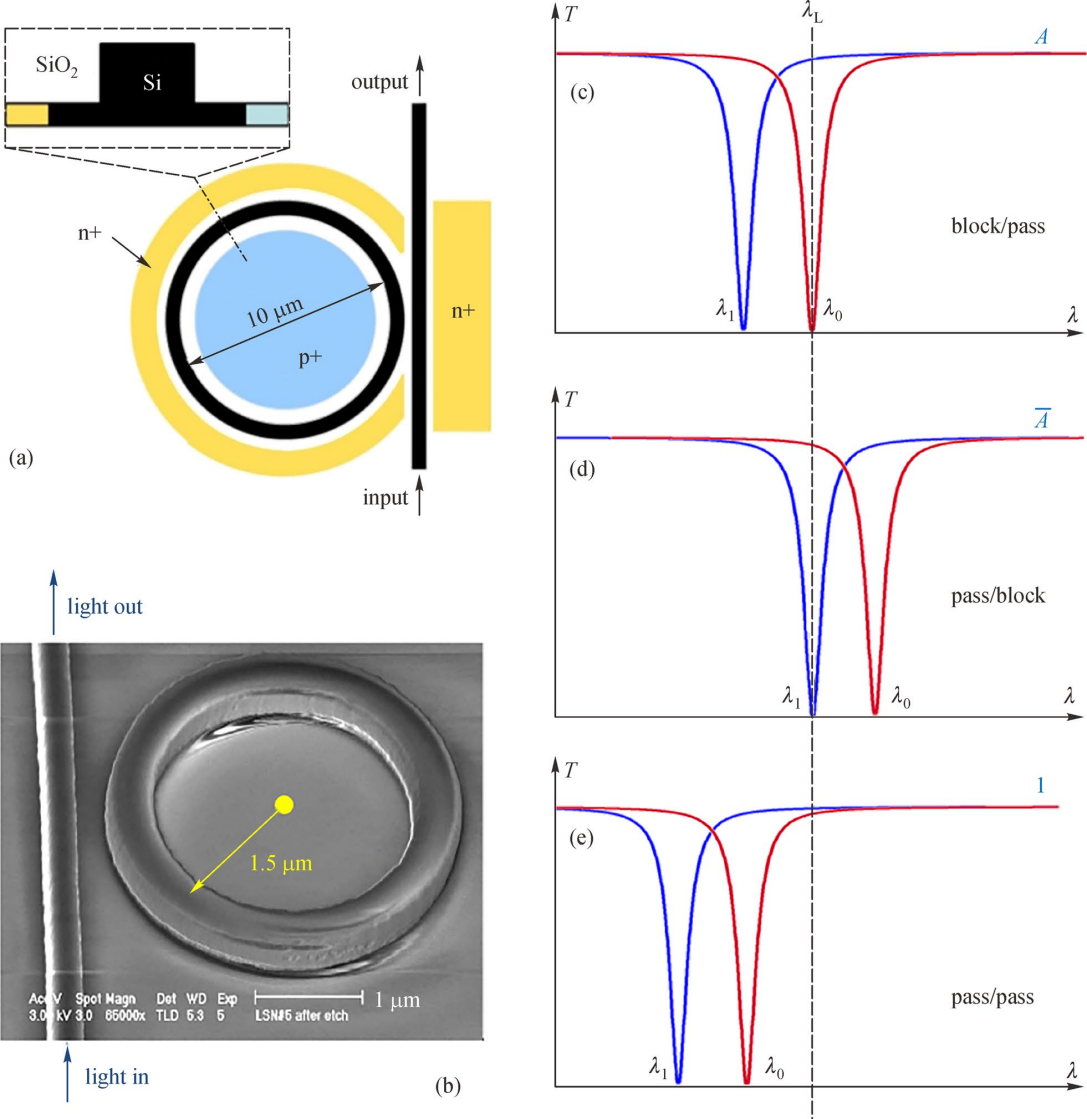
Structure and operation modes of a reconfigurable 1 × 1 switch.** a** Diagram of the switch. **b** SEM picture of a silicon MRR based switch. Here, a p-i-n junction is built across the ring. **c** Transmission spectra of a switch in ‘block/pass’ mode. Red line represents the spectrum when the logic signal is “1” with a resonant wavelength denoted as *λ*_1_. Blue line represents the spectrum when the logic signal is “0” with a resonant wavelength denoted as *λ*_0_. **d** Transmission spectra of a switch in “pass/block” mode. **e** Transmission spectra of a switch in “pass/pass” mode. Reprinted with permission from Ref. [93]. Copyright 2011, The Optical Society

In 2011, Prof. Qianfan Xu from Rice University and Prof. Richard Soref from the Air Force Research Laboratory proposed a reconfigurable electro-optic DL architecture that can calculate complex logic functions with only one chip [93]. In that paper, each optical switch consists of a micro-ring resonator, as shown in Fig. 7. By using the carrier plasma dispersion effect, the resonance wavelength can be controlled by electrical signal through integrated p-n or p-i-n junctions as shown in Fig. 7b. Note that the resonance wavelength can be denoted as *λ*_1_ when the electrical control signal is logic “1” and the resonance wavelength can be denoted as *λ*_0_ when the electrical control signal is logic “0”. Meanwhile, the wavelength of input light is *λ*_L_. Then, as shown in Fig. 7c, if *λ*_1_ matches *λ*_L_, light is blocked by the waveguide if the logic input is “1” and can pass through when the logic input is “0”. Thus, this operational mode is called the “block/pass” mode. Meanwhile, as shown in Fig. 7d, if *λ*_0_ is the same as *λ*_L_, light in the waveguide is blocked when the logic input is “0” and pass through the waveguide when the logic input is “1”. Thus, the operational mode is called “pass/block” mode. Moreover, if neither *λ*_0_ nor *λ*_1_ matches *λ*_L_, light can always pass through the waveguides, as shown in Fig. 7e. Then, this operational mode is called “pass/pass” mode.

In the above-mentioned three operational modes, the optical logic output depends on the electrical logic input “*x*”. For instance, in the “pass/block” mode, the logic output is the same as the logic input “*x*”. In the “block/pass” mode, the logic output is the inverse of the logic input. In the “pass/pass” mode, the logic output is “1”, which is independent of the logic input. Note that, for each switch, the operational modes can be reconfigured through the thermo-optic effect by using integrated micro-heaters. The reconfigurable speed would be ~ 20 kHz.

**Fig. 8:**
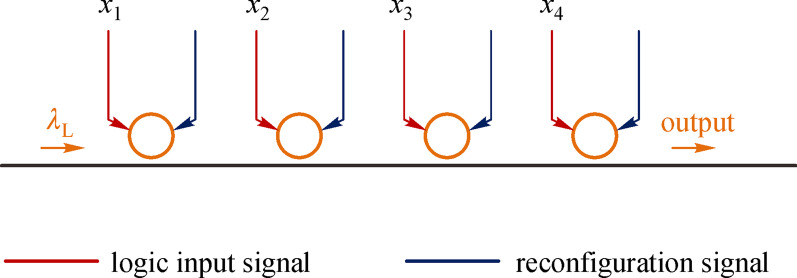
Layout and the electrical actuation for calculating the logic product. Here, each ring-based switch has a logic input signal and reconfiguration signal

With such reconfigurable operational modes of the switch, the optical logic Boolean product can be calculated. As shown in Fig. 8, several optical switches cascade onto the same waveguide. The *i*th switch has an electrical signal *x*_*i*_ for the logic input and the operational modes of the switch can be reconfigured by a thermal heater. Note that light can pass through the waveguide only when all the ring-based switches are in the “pass” state. Then, the final logic output of the waveguide is the product of all the electrical logic inputs for these switches. For instance, if the product ${x}_{1}{\overline{x} }_{4}$ is needed, one can set the first switch to operate in “pass/block” mode, the fourth switch to operate in “block/pass” mode, and the other switches to operate in “pass/pass” mode.

**Fig. 9:**
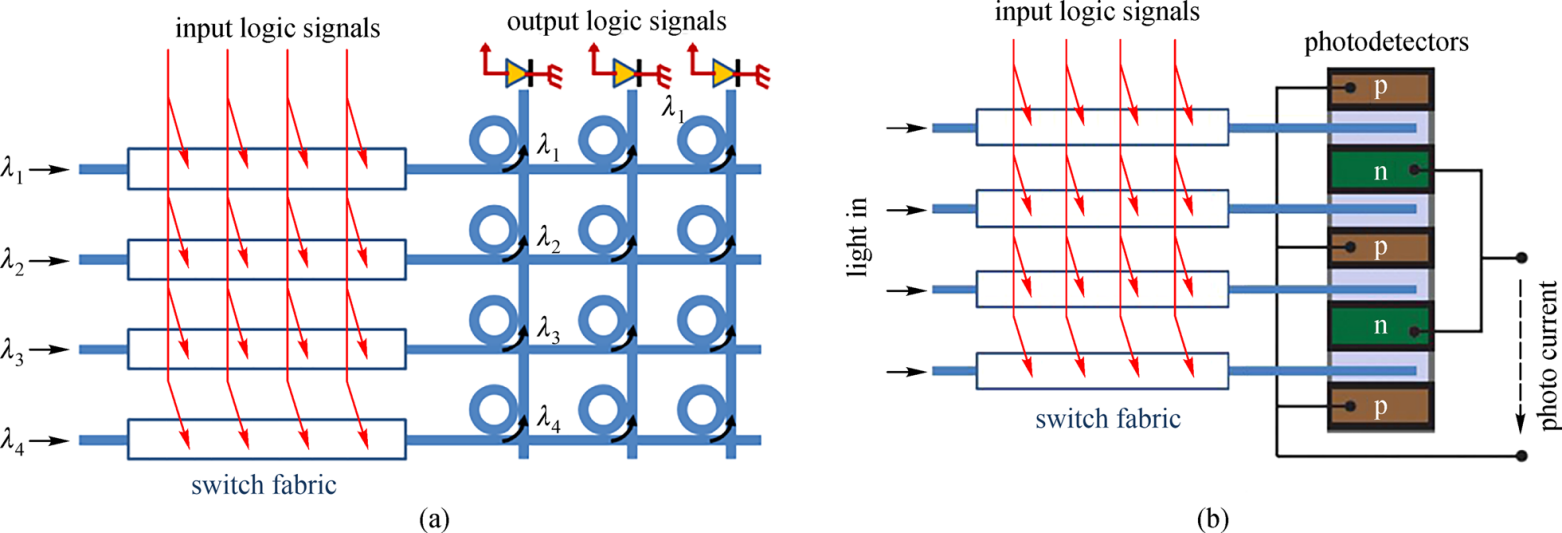
Diagrams of logic circuit. **a** Sum operation are performed by add-drop MRRs, which collect optical output at different wavelengths into one waveguide. **b** Sum operation are performed by parallel connected p-i-n photodetectors in the electrical domain. Reprinted with permission from Ref. [93]. Copyright 2011, The Optical Society

As is known to all, any binary logic functions can be expressed as a sum of product expressions. For example, $Y { = }y_{{1}} { + }y_{{2}} { + }y_{{3}} { + }...{ + }y_{n}$, in which *y*_*n*_ denotes the product of a number of variables *X*_*i*_, such as $y_{n} = \overline{{X_{1} }} \cdot X_{2} \cdot \overline{X}_{3} \cdot ... \cdot X_{m}$ ($\, \overline{X}_{m}$ represents the reverse logic value of *X*_*m*_). The product expression of *y*_*n*_ can be achieved by cascading several MRRs using the method mentioned above in Fig. 8. When calculating the sum expression, there are two approaches to eliminate the optical coherent issue. The first method, shown in Fig. 9a, is to use the optical wavelength division multiplexing (WDM) technique. In this method, different waveguides have different input wavelengths, thus the output of each horizontal waveguide has a logic product. One can then use a micro-ring resonator to direct the optical logic product to the output if the logic is used in the logic functions. Since different logic products are calculated using different wavelengths, the coherent issue can be limited. The second method is to utilize electrical splitter and PD arrays to obtain the sum expression, as shown in Fig. 9b. In this method, the optical product is first converted into electrical signals. Then, the electrical current from PDs can be added in the electrical domain to obtain the incoherent sum.

**Fig. 10:**
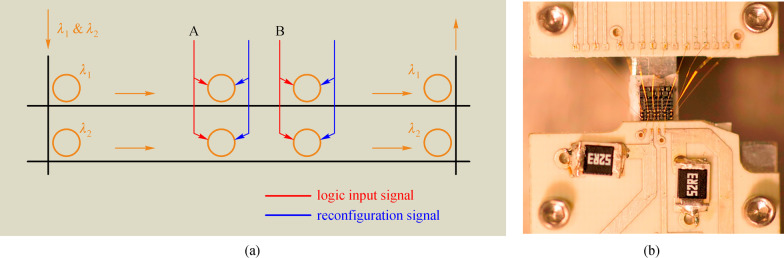
**a** Layout and the electrical actuation of the multi-spectral logic circuit. **b** Chip after packaging. Reprinted with permission from Ref. [95]. Copyright 2014, The Optical Society

For proof of concept, Dr. Ciyuan Qiu from Prof. Xu’s group of Rice University has implemented the above-mentioned ideas [94, 95]. A 2 × 2 logic array for two logic inputs was first demonstrated, as shown in Fig. 10 [95]. In that circuit, each switch consists of a micro-ring resonator with an embedded p-i-n junction and micro-heater. Then, for each switch, the electrical logic input can be applied through the p-i-n junction, while the operational mode of the MRR-based switch can be reconfigured using a thermal heater. The logic output is directed to the output through an add-drop ring. Based on this architecture, any two-input logic functions can be implemented with a speed of 500 Mb/s. In this case, the speed is limited by the response time of the p-i-n junction [94]. To further improve the speed, a new photonic circuit is implemented, where the p-i-n junction is replaced by a p-n junction [95]. In this circuit, the speed can approach 3 Gb/s, but is limited by the inductance of the wire-bonds and the capacitance of the p-n junction.

**Fig. 11:**
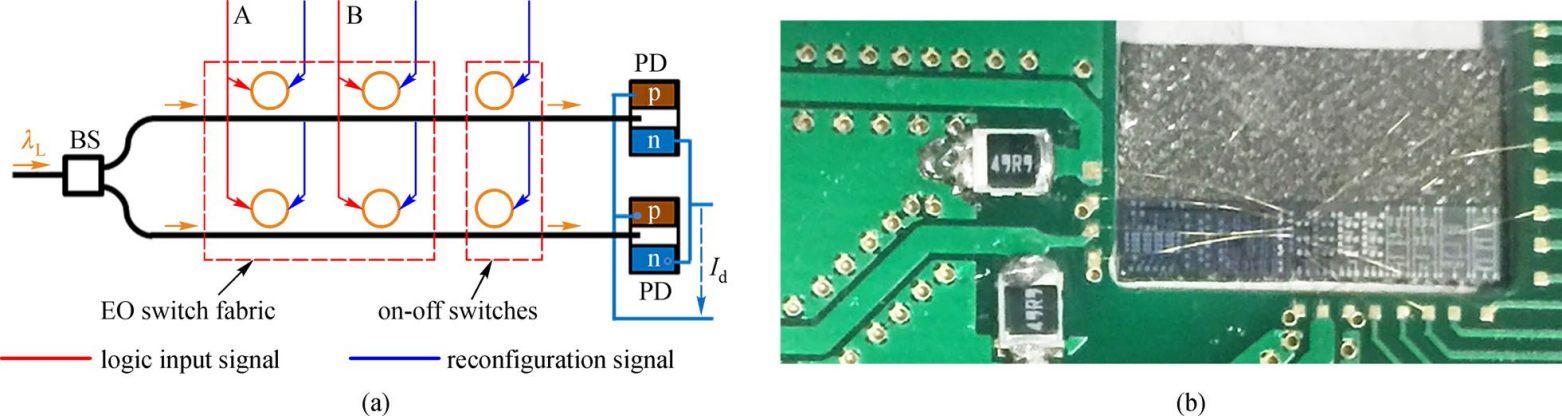
**a** Layout and the electrical actuation of the logic circuit with single light input. **b** Chip after packaging. Reprinted with permission from Ref. [96]. Copyright 2019, IEEE

The second approach for the logic computing is also demonstrated by Dr. Ciyuan Qiu [96]. As shown in Fig. 11, light input is equally divided into two parts using the beam splitter (BS) and subsequently sent to two waveguides. In each waveguide, there are two MRR-based EO switches and one ring resonator-based on–off switch. From the two switches, a two-input logic product can be calculated. If the logic input is used in the final function, then the on–off switch is on and light can pass through the waveguide. Otherwise, the on–off switch is in the “off” state and light is blocked and sent to PD to generate photocurrent. The photon currents from PDs can then be added together to obtain the incoherent sum. With such configuration, a two-input logic function with single wavelength input is implemented with a speed of 270 MHz. Here, the speed is limited by the switch’s carrier injection mode that is needed to reduce the RF noise, which has a strong influence on the PD.

**Fig. 12:**
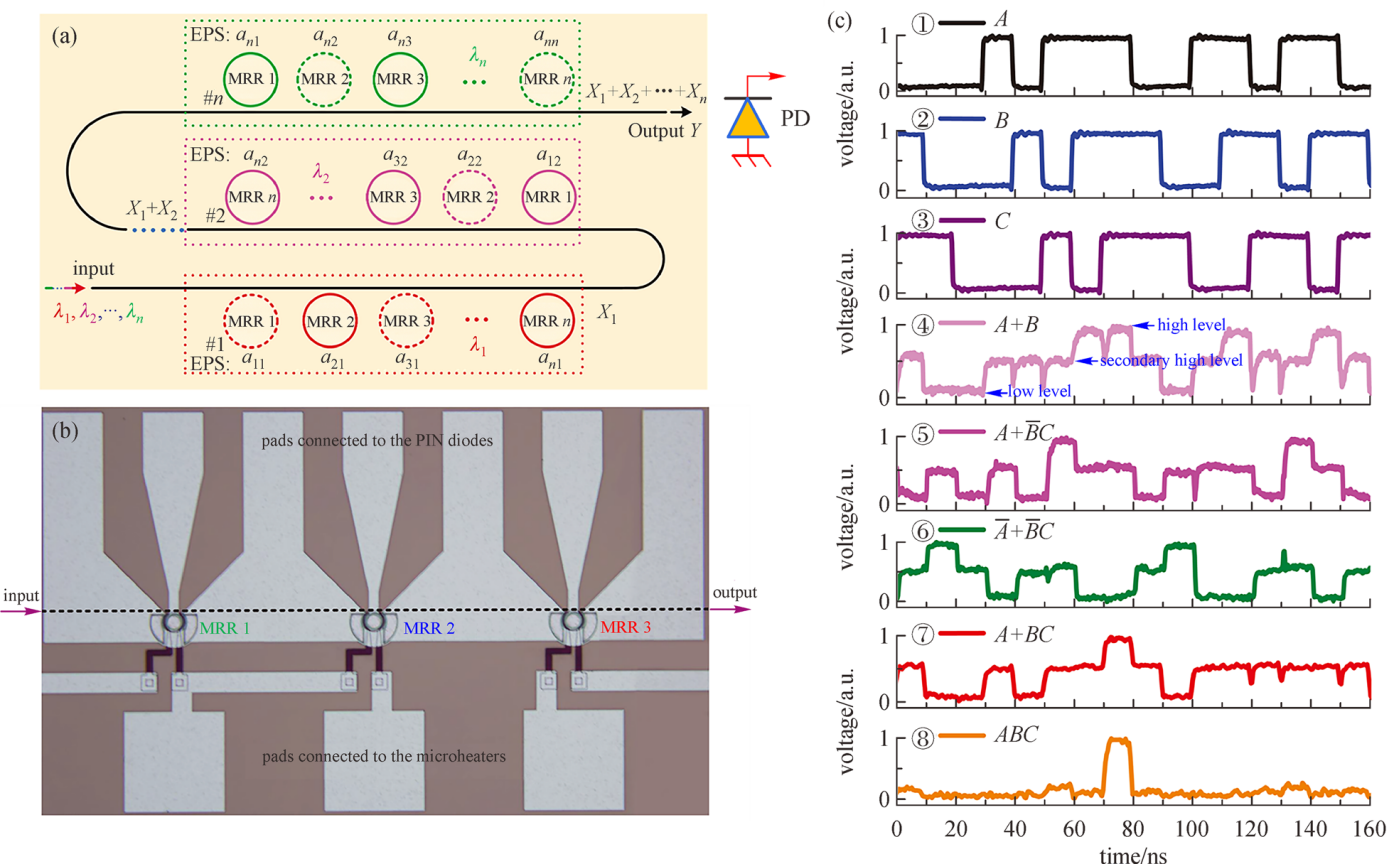
DL circuit composed of cascaded MRRs. **a** Schematic structure and **b** micrograph of the fabricated device for concept proof. **c** Measured dynamical computing result. **a** Reprinted with permission from Ref. [97]. Copyright 2016, IEEE. **b** and **c** Reprinted with permission from Ref. [98]. Copyright 2017, Springer Nature

In 2016, a new structure to perform reconfigurable logic computing was proposed and demonstrated [97], as shown in Fig. 12a. Compared to the two approaches mentioned above, this structure is much simpler since the wavelength demultiplexers, multiplexers, and PD arrays are all eliminated. Only a single waveguide and MRR array are utilized in this structure. The cascaded MRRs are first divided into several groups, each with one independent working wavelength. For one group, the working principle is the same as the block described in Fig. 10, with each MRR reconfigured to work in one of the three modes: pass/block, block/pass, or pass/pass. The number of MRRs and working wavelengths in each group can also be reconfigured. Straight waveguides are adopted to connect different groups. The input light is with several wavelengths as the computing groups desired and the output signals are directly fed to a wideband PD for result observation. A device was designed and fabricated based on p-i-n junctions embedded MRRs [98], as shown in Fig. 12b. The measured results shown in Fig. 12c indicate that the fabricated device can be reconfigured to implement various logic functions with a speed of 100 Mbps. A high computing speed can be expected when utilizing reverse-biased p-n junctions for modulation.

## Low-power DL computing

Power consumption is another issue that should be taken into consideration in the logic computing. Note that with *N*-input logic functions, there are 2^*N*^ different logic products. To implement each logic product, *N* optical switches are needed. Then, the number of the power computing *P*_total_ could be expressed as *P*_total_ ~ *N* × 2^*N*^*P*_unit_ for a reconfigurable circuit to perform *N*-input logic functions. Here, *P*_unit_ is the dynamic power for each unit switch in the computing process. Meanwhile, if the logic function is fixed, *P*_total_ can be expressed as *P*_total_ ~ *M* × *P*_unit_, where *M* is the number of switches needed in the functions. Based on the discussion above, we can see that one can decrease *M* or reduce the unit power (*P*_unit_) in order to efficiently reduce the total power consumption of the circuit.

**Fig. 13:**
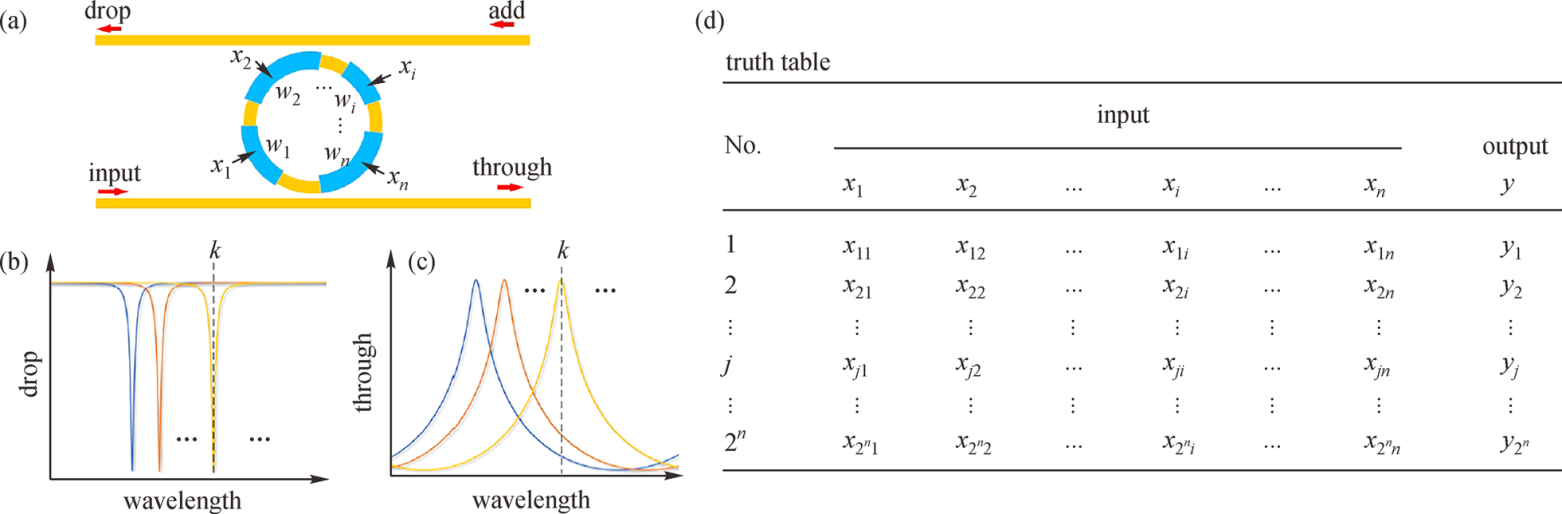
Principle of multi-operands logic gate (MOLG). **a** General MOLG with weighted inputs. **b** Transmission spectrum at the drop port. **c** Transmission spectrum at the through port. **d** Truth table. Reprinted with permission from Ref. [99]. Copyright 2019, the American Institute of Physics

To reduce the number *M* for logic function, Prof. Ray Chen’s group at the University of Texas-Austin tried to implement logic functions with only one switch [99]. As shown in Fig. 13, they proposed and demonstrated a micro-ring based multi-operand logic gate (MOLG). In their work, each ring had multiple operands (denoted as *x*_1_–*x*_*n*_), each controlling one active segment of the ring modulator. The logic input applied to the active segment *i* with the weight of *w*_*i*_ is marked as *x*_*i*_. It can be assumed that applying the electrical control signal result in a redshift of the transmission spectrum. Then, as shown in Fig. 13b and c, the redshift accumulates with multiple inputs. The truth table for the output *y* could be found in Fig. 13d. Note that each operand could have two states: “0” or “1”, thus the truth table would be 2^*n*^ rows for different logic functions. Meanwhile, each output *y*_*i*_ also has two states so any combination of *y*_*i*_ along with 2^*n*^ input rows represents a logic function. With such setup, the logic function *y*_*i*_ could also be reconfigured by tuning the initial wavelength of the switch with a thermal heater. In this method, the power consumption can be effectively reduced since only one switch is used to implement the logic functions.

**Fig. 14:**
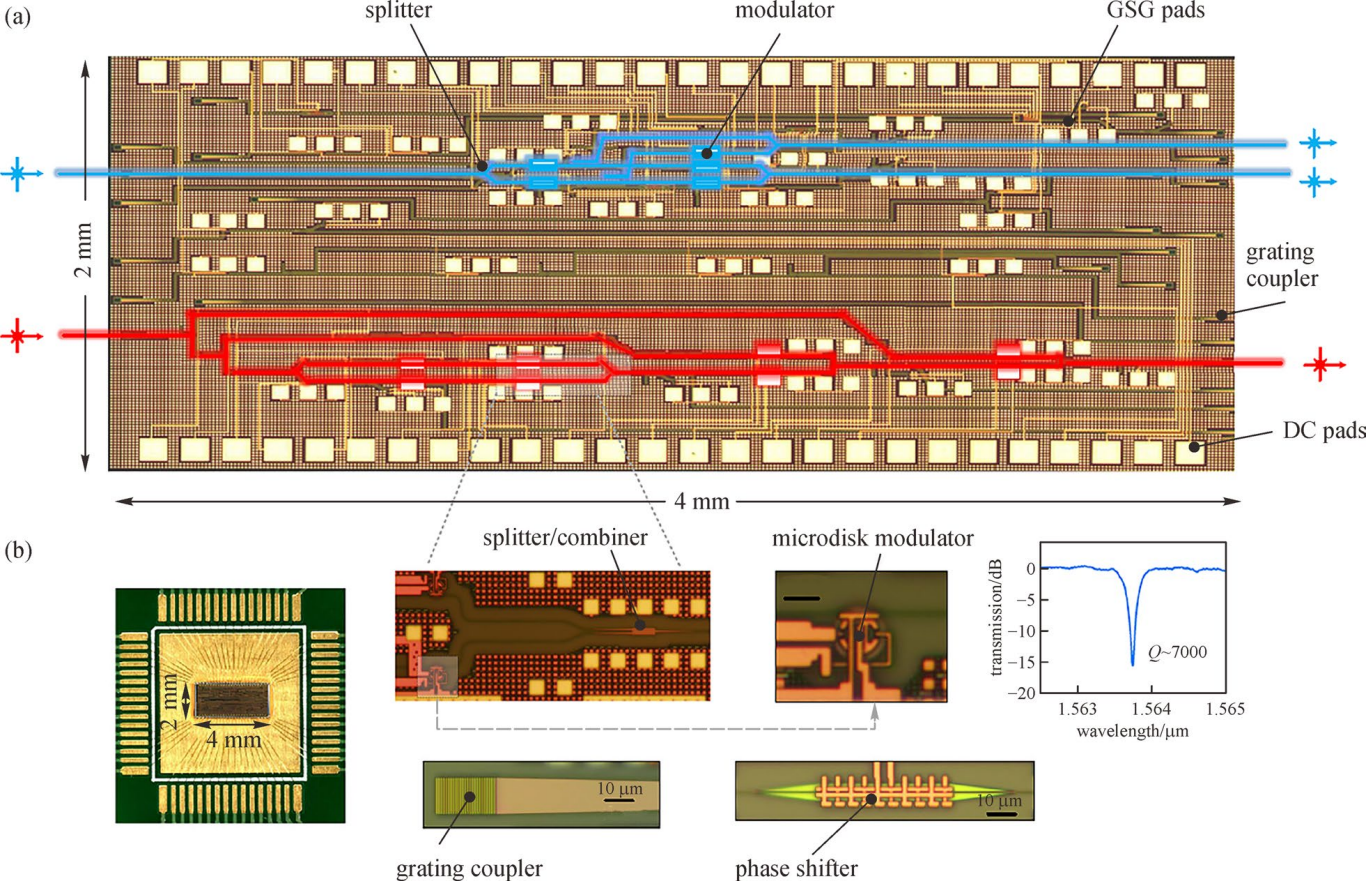
Experimental demonstration. **a** Optical micrograph illustration of the chip. **b** Micrographs of the wire bonded chip. Fundamental photonic components such as the splitter/combiner, the phase shifter, the grating coupler, and the microdisk modulator are included. Here, the spectrum of one peak of the microdisk is also shown. Reprinted with permission from Ref. [104]. Copyright 2020, Springer Nature

Another way to reduce power consumption is by decreasing the unit power of the switches. To achieve this, Prof. Chen’s group also explored the application of microdisk switches. In Refs. [100, 101], a comparison was first comprehensively discussed between the MRR-based and MDR-based switches in terms of dimensions, power consumption, and fabrication tolerance. Since the micro-disk has a smaller footprint and lower capacity (*C*), the dynamic power (~ *CV*^2^/4, where *V* is the voltage applied on the switch) can be significantly decreased for MDR-based switches. Thus, a MDR-based switch array has significant advantages over MRR-based switch fabrics in logic computing in terms of footprint and power consumption. In 2018, they demonstrated a proof of concept for silicon micro-disk-based adders [102, 103]. In those works, the microdisk had a radius of 2.5 μm with a 100 nm gap between the microdisk and bus waveguide. Such radius is much smaller than that of a micro-ring resonator. The adder applications are realized with a speed ~ 2.56 Kbps. It is important to note that such speed is limited by the fact that an electrical logic input signal is applied onto the switch through thermo-optic tuning. To further improve the speed and reduce the power consumption, they recently experimentally demonstrated a 4-bit arithmetic logic unit with an operational speed of 20 GHz [104]. In that work, the unit consists of 8 high-speed microdisk optical switches, as shown in Fig. 14. Meanwhile, the power consumption per bit of the switch is estimated to be 10.88 fJ/bit, with a 0.8 V swing voltage and an approximate 17 fF microdisk capacitance. Note that this power is much lower than the 300 fJ/bit of the MRR-based switches [95]. Thus, such architecture could be a good candidate to calculate complex logic functions.

## Programmable photonic networks for DL

**Fig. 15:**
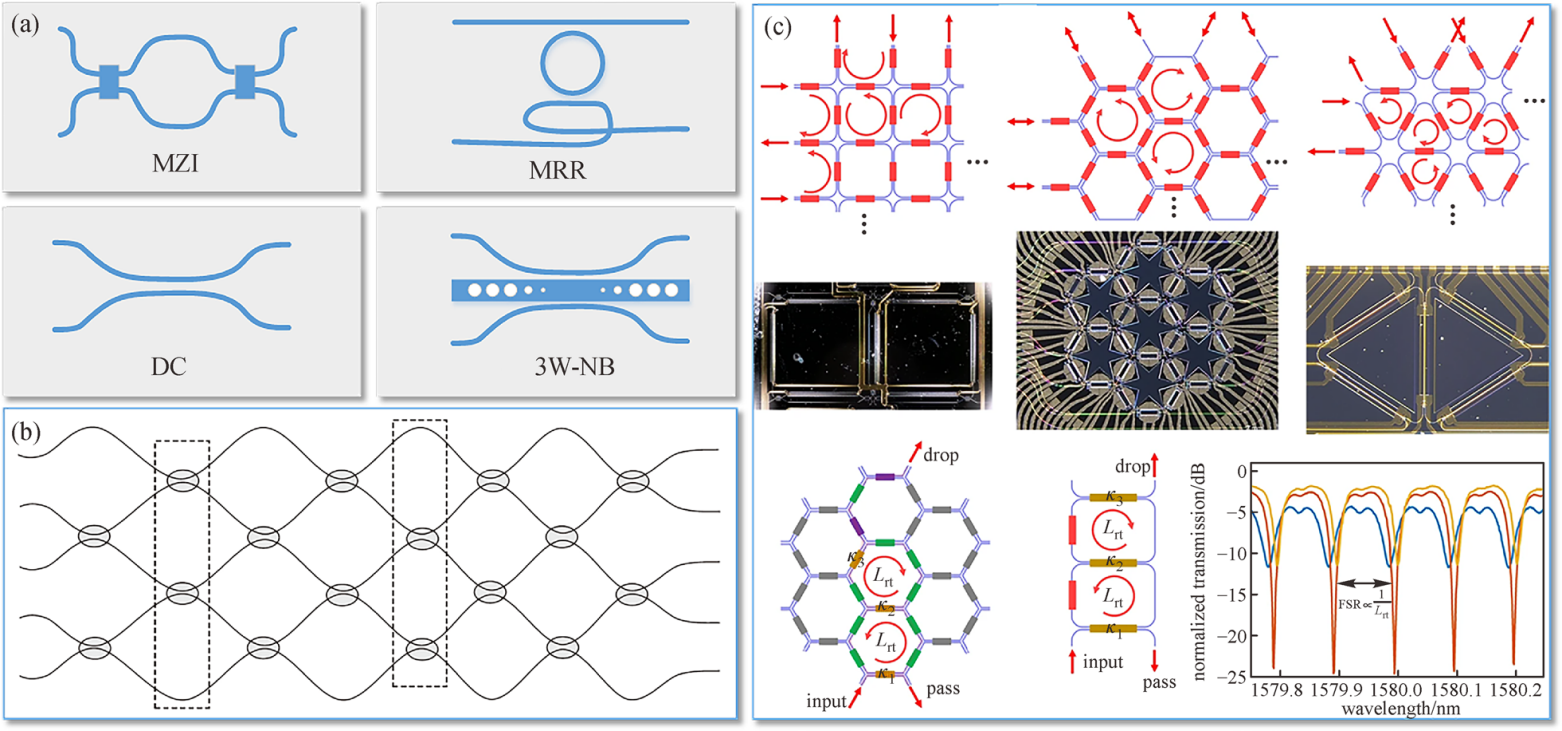
Structure of programmable optical logic networks. **a** 2 × 2 building blocks of optical network (MZI: Mach–Zehnder interferometer, MRR: Micro-ring resonator, DC: Directional coupler, 3W-NB: optical switch based on three waveguides with central nanobeam). **b** Forward only mesh network, each circle is a 2 × 2 optical switch, and each dashed rectangle delineates a single switching layer. **c** Recirculating waveguide meshes based on square cells, hexagonal cells, and triangular cells, each small rectangles represent a 2 × 2 optical switch, the green, purple, yellow and red rectangles indicate that the corresponding switch is working in a bar state, cross state, partial coupling state, and arbitrary state, respectively. **b** Reprinted with permission from Ref. [108]. Copyright 2013, Elsevier. **c** Reprinted with permission from Ref. [105]. Copyright 2020, Springer Nature

Returning to Fig. 1, the final destination of DL is to utilize an optical switching network to implement large-scale, low power, and high-speed optical computing. The above reviewed reconfigurable logic circuit and low-power optical switches provide an efficient solution. Programmable photonics, which aim to design common photonic networks to implement various basic and complex functionalities in many application fields, offer an alternative method to implement reconfigurable large-scale and high-speed optical logic computing. The distinguished properties of programmable photonic networks include flexible reshaping of finite resources, robustness and resilience, creating systems quickly and infinite resources through timesharing [105]. Basically, the programmable photonic network is formed by cascading a large amount of 2 × 2 building blocks including the MZI, MRR, directional coupler (DC), optical switch based on three waveguides with central nanobeam [106, 107], etc. The typical topological structures of 2 × 2 building blocks and programmable photonic networks are presented in Fig. 15.

In 2013, Prof. Joseph Shamir proposed a generic programmable optical network, as shown in Fig. 15b, to perform reconfigurable and reversible logic operations [108]. Within the DL paradigm, one can supply a light input vector with at least one nonzero element and the result of a logic operation can be detected in the output ports. The electrical operands can be applied to the switches or phase shifters as desired. The number of switches or phase shifters taking effect and their working states can be reconfigured to implement different logic functions. One basic property of this kind of network is that it is a forward only mesh network, meaning the computed logical result can only be obtained on one side since light can only flow from the input to the other side. This phenomenon is the reason behind the simple progressive setup of forward only mesh networks and allows the system to minimize or maximize the power of photodetectors, or in some cases, self-configure to specific problems or self-stabilizes its operation [109–111], all of which are efficient for advanced DL computing applications, such as matrix processor [112, 113] and optical convolution [114].

Programmable photonic networks can also be achieved though network recirculation [115]. Figure 15c lists three kinds of recirculating network structures based on square cells, hexagonal cells, and triangular cells, respectively. These cell types allow the recirculating networks to have loop structures, making it possible for light in the network to be routed in any direction or even back to the input ports, which is very effective for optical artificial neural networks [116]. Within the DL paradigm, the electrical operands in recirculating network can be *M*-ary to control the working states of the switches (bar state, cross state, or partial coupling state) [115], making the recirculating network promising to implement *M*-ary DL computing based on the principle described in Sect. 2.1. Thus, the programmable photonic network can be more comprehensive and versatile to realize optical computing, signal filtering, waveform generating, reconfigurable delaying, optical beamforming, and many other functionalities simultaneously or separately.

## Conclusion

Optical directed logic (DL) computing plays a key role in the field of high-speed optical information processing. In this paper, we reviewed the current development and potential trend of integrated optical DL on SOI platform. Various works have been demonstrated from fundamental logic gate to combinational DL since the first proposal of DL in 2007. During the development of DL, the continuing improvement of high-speed modulation techniques and low switching-power optical switches impel DL to become an increasingly efficient and competitive method for optical computing. For future multi-bit and *M*-ary large-scale computing, the schemes of reconfigurable DL and programmable photonic network are believed to be promising candidates. In the near future, the integration of DL and large bandwidth PD in the same circuit is more likely to pave the way for the next-generation “electronic-photonic digital computer”.
